# Supplementary material for: Training intervention to improve hygiene practices in Islamic boarding school in Yogyakarta, Indonesia: A mixed-method study
Source: PLoS One. 2020 May 29;15(5):e0233267. doi: 10.1371/journal.pone.0233267 (PMC7259706; doi:10.1371/journal.pone.0233267)
Supplement: S1 Appendix — (DOCX) [file pone.0233267.s001.docx]

| **Date of Assessment** |
| --- |
| **__ __ / __ __ / __ __ __ __** |

| **Identification Number** | **Initial** | **Type of Questionnaire** |
| --- | --- | --- |
| __--__ __--__ __ __ | __ __ __ | **Reaction** |

Circle the appropriate assessment.

| **No.** | **Criteria** | **Apparisal** | | | | |
| --- | --- | --- | --- | --- | --- | --- |
| 1. | Benefits of training | 1 | 2 | 3 | 4 | 5 |
| 2. | Training materials | 1 | 2 | 3 | 4 | 5 |
| 3. | The training process | 1 | 2 | 3 | 4 | 5 |
| 4. | Satisfaction with the training management process | 1 | 2 | 3 | 4 | 5 |
| 5. | Satisfaction with coach | 1 | 2 | 3 | 4 | 5 |

Description: 1 = very bad  4 = good

2 = not good   5 = excellent

3 = fairly

**Page 1**

| **Date of Assessment** | **Pre Test ___** |
| --- | --- |
| **__ __ / __ __ / __ __ __ __** | **Post Test**  ___ |

| **Weeks** | | | | | | | |
| --- | --- | --- | --- | --- | --- | --- | --- |
| **1** | **2** | **3** | **4** | **5** | **6** | **7** | **8** |

| **Identification Number** | **Initial** | **Group** | **Type of Questionnaire** |
| --- | --- | --- | --- |
| __--__ __--__ __ __ | __ __ __ | **1 / 2 / 3** | **Knowledge** |

Circle the appropriate assessment.

| **No.** | **Criteria** | **Appraisal** | |
| --- | --- | --- | --- |
| 1. | Cut nails and keep them clean indicate good hygiene | Correct | Wrong |
| 2. | Maintain cleanliness of hair is part of personal hygiene | Correct | Wrong |
| 3. | Wash your hair regularly is part of personal hygiene | Correct | Wrong |
| 4. | Washing hands with soap regularly as part of personal hygiene | Correct | Wrong |
| 5. | Hand washing can prevents transmission of flu | Correct | Wrong |
| 6. | Biting fingernails can damage dental health | Correct | Wrong |
| 7. | The clean rooms can prevent skin infections | Correct | Wrong |
| 8. | The clean rooms can prevent cough | Correct | Wrong |

**Page 2**

| **Date of Assessment** | **Pre Test ___** |
| --- | --- |
| **__ __ / __ __ / __ __ __ __** | **Post Test**  ___ |

| **Weeks** | | | | | | | |
| --- | --- | --- | --- | --- | --- | --- | --- |
| **1** | **2** | **3** | **4** | **5** | **6** | **7** | **8** |

| **Identification Number** | **Initial** | **Group** | **Type of Questionnaire** |
| --- | --- | --- | --- |
| __--__ __--__ __ __ | __ __ __ | **1 / 2 / 3** | **Behavior** |

Circle the appropriate assessment.

| **No.** | **Criteria** | **Appraisal** | |
| --- | --- | --- | --- |
| 1. | I shower twice a day | Yes | No |
| 2. | My hair combed least once a day | Yes | No |
| 3. | I brush my teeth every morning and before bed | Yes | No |
| 4. | I wash the hair at least twice a week | Yes | No |
| 5. | I clipped nails once a week | Yes | No |
| 6. | I wash my hands with soap at least 20 seconds before and after meals | Yes | No |
| 7. | I wash my hands with soap at least 20 seconds after the toilet | Yes | No |
| 8. | I shower after physical activity or sport sweat | Yes | No |
| 9. | I change underwear minimum once a day | Yes | No |
| 10. | I use clean clothes every departing activity | Yes | No |
| 11. | I tidied up the mattress before going to school | Yes | No |
| 12. | I put dirty clothes in place | Yes | No |
| 13. | I dispose of waste in place | Yes | No |
| 14. | I wash utensils that have been used immediately after completion use | Yes | No |
| 15. | I left the room in a tidy state before going to school | Yes | No |
| 16. | I change the sheets at least two weeks | Yes | No |

**Page 3**

| **Date of Assessment** | **Pre Test ___** |
| --- | --- |
| **__ __ / __ __ / __ __ __ __** | **Post Test**  ___ |

| **Weeks** | | | | | | | |
| --- | --- | --- | --- | --- | --- | --- | --- |
| **1** | **2** | **3** | **4** | **5** | **6** | **7** | **8** |

| **Identification Room Number** | **Group** | **Type of Questionnaire** |
| --- | --- | --- |
| __--__ __ | **1 / 2 / 3** | **Outcome** |

Circle the appropriate assessment.

| **No.** | **Criteria** | **Appraisal** | |
| --- | --- | --- | --- |
| 1. | Clean floor | Yes | No |
| 2. | Neat mattress | Yes | No |
| 3. | No food leftovers on the floor | Yes | No |
| 4. | No dirty clothes in bed | Yes | No |
| 5. | There is no garbage strewn | Yes | No |
| 6. | Goods regulated according to its kind | Yes | No |
| 7. | Clothes neatly in the closet | Yes | No |
| 8. | The books are neatly arranged in shelves | Yes | No |
| 9. | Daily needs in order neatly under the bed | Yes | No |
| 10. | Footwear neat | Yes | No |

**Page 4**
