# Supplementary material for: Training intervention to improve hygiene practices in Islamic boarding school in Yogyakarta, Indonesia: A mixed-method study
Source: PLoS One. 2020 May 29;15(5):e0233267. doi: 10.1371/journal.pone.0233267 (PMC7259706; doi:10.1371/journal.pone.0233267)
Supplement: Attachment — Submitted filename: Reviewer Plos One.docx [file pone.0233267.s003.docx]

Dear Authors

The manuscript entitled "TRAINING INTERVENTION TO IMPROVE HYGIENE PRACTICES IN ISLAMIC BOARDING SCHOOLS IN YOGYAKARTA, INDONESIA: A MIXED METHODS STUDY" is very important issues of health and hygiene in school health areas. However, some problematic issued of writing error for scientific paper in this manuscript is identified.

1. Abstract

- In the method, please mention how may islamic boarding school attended in this study? and how to combine for analysing mixed method for qualitative and quantitative data in this study?

- Results: The authors just mention qualitative and quantitative data separately. Please show the mixed method results from this study?

- Conclusion: It is too shortly just from quantitative data analysis. Please re-statement from your thesis statement.

2. Introduction

The authors have mentioned the problems of this study, solution for this study and their outcomes. However, before the last paragraph, the authors was missed some issues of this study. Therefore, before your objective the study, please describe more the characteristic of lifestyle of Islamic boarding schools (IBS) regarding Islamic perspective and schools health perspective. Why the authors choose mixed method for solution in this study, therefore the authors should explore more phenomena of Santris in IBS.

3. Method

a. On study site:

- Please describe what is Salafi? This research just one site? Or Multi centers study?
- How many sample size and what is your sampling technique?

b. Study design

- It is not clearly. Please describe more how many sample in qualitative study? Who is supervisors in this study?
- In quantitative study, who is students? How the authors collected the student?
- What is inclusion and exclusion criteria in this study? Supervisor? Student?

c. Data collection and analysis * qualitative

- It is very confused. In the abstract the authors mentioned three and one FGD, although in this section, they said two FGD, which ones right?
- Please describe the supervisors attended in your study?

d. Data collection and analysis * quantitative

- What is your criterion eligibility of sample in this study?
- How you allocated in this study?

e. I think its better you should describe in one different parts about your Intervention study. Please make one sub heading in method which mentioned about your intervention study? Why the authors want to looks the differences between boys and girls? There was no reason that they mentioned in introduction if the boys and girls is differences on their hygiene?
f. Please describe more your instruments for quantitative study, how about the validity and reliability? It is in Bahasa Indonesia version? Please explain how many questions?
g. Training intervention.

- Regarding first meeting FGD with supervisor, how the authors conducted the training? It is something missing?
- Please describe more clearly your Figure 2, regarding your first meeting FGD with supervisor?

3. Result

- On abstract the authors mentioned 20 participants for qualitative, although in this section they mentioned 15 of total 21? Which ones right?
- The authors should mentioned qualitative results for the first study to measure themes for material education health training
- Then, they should interview again regarding after training ended and after the study finished?
- Regarding table 2. Before intervention study, regarding training perception (benefits training, training process, satisfaction training and coach) there are differences both of genders. Therefore, from the starting of study based on gender the starting point is not homogeneity. Why the authors analyse based on the gender in this study?
- Please show us the characteristics of participants students to compare their starting point before study?
- Why the authors compared between boys and girls, because there are differences before training? (not homogeneity)

4. Discussion

- Please re-write based on your objective your study?

- What is the good and what is the bad? Please write based on your mixed method results?

5. Conclusion

Re-write your thesis statement?

6. references

Please re-write based on authors guideline

Thank you.

Sincerely yours,

Reviewer
