# Supplementary material for: Training intervention to improve hygiene practices in Islamic boarding school in Yogyakarta, Indonesia: A mixed-method study
Source: PLoS One. 2020 May 29;15(5):e0233267. doi: 10.1371/journal.pone.0233267 (PMC7259706; doi:10.1371/journal.pone.0233267)
Supplement: Attachment — Submitted filename: Response_academic editor_final.docx [file pone.0233267.s005.docx]

Response to the Academic Editor, Prof. Soundy:

Thank you for providing the opportunity to revise our manuscript. Here is our response:

1. When submitting your revision, we need you to address these additional requirements.

Please ensure that your manuscript meets PLOS ONE's style requirements, including those for file naming. The PLOS ONE style templates can be found at

<http://www.journals.plos.org/plosone/s/file?id=wjVg/PLOSOne_formatting_sample_main_body.pdf> and <http://www.journals.plos.org/plosone/s/file?id=ba62/PLOSOne_formatting_sample_title_authors_affiliations.pdf>

Our response:

Thank you for the reminder. We have revised the format according to the template style.

2.  Please refer to any post-hoc corrections to correct for multiple comparisons during your statistical analyses. if these were not performed please justify the reasons. Additionally, in the text you state: "The subjects were nonrandomly assigned to one of three different interventions". Please discuss how this assigning occurred.

Our response:

Thank you for addressing this important point. For the analysis on knowledge and behavior, we did the post hoc analysis and described the result. But, we did not used the post hoc test for room hygiene analysis because the finding from the Kruskall-Wallis test was not statistically significant. The study used a quasi-experimental study, i.e. a stepped wedge pre-post analysis and in the method section, we have explicitly stated that the allocation of participants was nonrandom.

3.  Please provide further details concerning the piloting of the interview guide used in this study. I.e. who were the participants and how many were they.

Our response:

Thank you for addressing this. We did not pilot the interview guide in this study, because the interview was only conducted with the school principal. However, prior to the qualitative data collection activities, the first author spent six months to get familiarize with the setting and build relationships with this boarding school.

4.  Please remove your figures from within your manuscript file, leaving only the individual TIFF/EPS image files, uploaded separately.  These will be automatically included in the reviewers’ PDF.

Our response:

Thank you for this reminder. We have removed the figures from the manuscript and will upload them separately.

Thank you,

Sincerely Yours,

Authors
