# Supplementary material for: Training intervention to improve hygiene practices in Islamic boarding school in Yogyakarta, Indonesia: A mixed-method study
Source: PLoS One. 2020 May 29;15(5):e0233267. doi: 10.1371/journal.pone.0233267 (PMC7259706; doi:10.1371/journal.pone.0233267)
Supplement: Attachment — Submitted filename: 2nd COMMENTS TO AUTHORS_Plos One.docx [file pone.0233267.s006.docx]

**COMMENTS TO AUTHORS**

I do not see any of my earlier comments addressed by the authors. I resubmit them as follows:

**Abstract**

*“The primary objective of this study was to measure the impact of a training intervention on improving hygiene practices among Islamic boarding school students…”.*

Once again, I find it necessary for authors to replace the word “impact”. It is difficult for authors to convince readers that they truly measured impact. On the measurement chain, impact is in the very long term sometimes 10 years and more. This cannot be practicable for a hand hygiene study of this nature. They may consider outcome or effect instead.

**Introduction**

*In a 2014-2015 school health report, 70% of santris in a boarding school 92 were diagnosed with infectious diseases, predominantly respiratory and skin infections..”*

Cite the source document appropriately.

**Materials and Methods**

***Study Design***

This section needs some clarity. First of all;

i. what constituted your clusters?
ii. How did you control for clustering in considering a sample size?
iii. What was the unit of analysis?
iv. How did you decide on the number of students to participate in the study? For eg, did you estimate the figure statistically?

**Data Collection and analysis**

***Qualitative study***

i. How did you derive the 21 supervisors?
ii. What is the source of your data collection tools?

***Quantitative study***

i. What was the source of quantitative data collection tool?
ii. You mentioned the use of a Likert scale. How did you test for the internal reliability of items assessing the same variable?
iii. How did you control for clustering at the data analysis stage?

**Training intervention**

i. How did you ensure that training was the same for the different intervention groups?
ii. Did you assess the intervention fidelity to ensure that the interventions delivered were in adherence to the protocols of the study?

**Results**

It appears you statistically compared the outcomes within the boys groups with the outcomes within the girls group at some point. Such a comparison will require unpaired samples t-test and not a paired samples t-test.
