# Supplementary material for: Training intervention to improve hygiene practices in Islamic boarding school in Yogyakarta, Indonesia: A mixed-method study
Source: PLoS One. 2020 May 29;15(5):e0233267. doi: 10.1371/journal.pone.0233267 (PMC7259706; doi:10.1371/journal.pone.0233267)
Supplement: Attachment — Submitted filename: Response to Reviewer 4.docx [file pone.0233267.s007.docx]

**COMMENTS TO AUTHORS**

Let me congratulate authors for taking time to research into such an important phenomenon. I find the following comments useful for enriching the content of the manuscript.

Our response:

We really appreciate your further comments and we tried our best to provide the responses to improve the manuscript.

**Abstract**

*“The primary objective of this study was to measure the impact of a training intervention on improving hygiene practices among Islamic boarding school students…”.*

I caution authors against the use of the word “impact”. It is difficult for authors to convince readers that they truly measured impact. On the measurement chain, impact is in the very long term sometimes 10 years and more. This cannot be practicable for a hand hygiene study of this nature.

Our response:

Thank you and we fully agreed to this. We, therefore, have changed the term “impact” into “effect”. Taking into consideration comments from other reviewers, we then modified the primary objective of the study into study purpose and objectives, as follows.

Original sentence:

The primary objective of this study was to measure the impact of a training intervention on improving hygiene practices among Islamic boarding school students (*santris*).

Revision:

“The primary objective of this study was to determine the effect of a training intervention in overall improvement in students’ *(santris)* knowledge, behavior, and outcome.”

**Introduction**

*In a 2014-2015 school health report, 70% of santris in a boarding school 92 were diagnosed with infectious diseases, predominantly respiratory and skin infections..”*

Cite the source document appropriately.

Our response:

Thank you for your suggestion. We obtained this data from the Mawar Islamic Boarding School pilot study. We have revised the sentence to avoid misunderstanding as follow:

“In the 2014-2015 Mawar Islamic Boarding school’s health report, 70% of santris were diagnosed with infectious diseases, predominantly respiratory and skin infections”

**Materials and Methods**

***Study Design***

This section needs some clarity. First of all;

i. what constituted your clusters?

Our response:

Thank you for clarifying this. The intervention was conducted based on cluster of rooms. One room is occupied by 10-12 santris. A cluster is defined as a block of adjacent rooms. The number of rooms per cluster was 8 rooms for girls and 6-7 rooms for boys. The total number of rooms for girls and boys in our intervention was 24 and 20, respectively.

ii. How did you control for clustering in considering a sample size?

Our response:

Thank you for your question. We are aware of the trial sample technique for the Random Control Cluster. However, in this study, we did not estimate the sample size because we used the total population.

iii. What was the unit of analysis?

Our response:

The unit of analysis for reaction, knowledge, personal hygiene was individual person (in this case, santri), while for the room hygiene, the unit of analysis was room.

iv. How did you decide on the number of students to participate in the study? For eg, did you estimate the figure statistically?

Our response:

Thank you for this question. We involved all new junior high school students in the Mawar Islamic Boarding School.

**Data Collection and analysis**

***Qualitative study***

i. How did you derive the 21 supervisors?

Our response:

Thank you for this comment. Clarification on the supervisors was also asked by other reviewers. In a boarding school, supervisor is a senior student chosen by the school to take the role of supervisor. This designated santri supervisors (male and female) have at least completed senior high school from this school and they are chosen based on their high performance for the standard on religious practice and competence. Once they become supervisors, they have to live in the boarding school and are responsible to take care of 30-50 santris’, teach the Qur’an while undertaking their own activity, such as working as school staff or studying at university level.

In this study, we involved 20 supervisors and one school principal. The selection of the 20 supervisors was carried out in collaboration with the school owner to appoint those who have experiences in supervising new, first grade junior high school student. Out of 45 junior high school supervisors, only 20 supervisors met the criteria.

The above sentences have now been added in the section on data collection and analysis, page 6, line 138-141 as the following:

Original sentence:

“The participants in the qualitative study were twenty-one santri supervisors. Data were collected through three focus group discussions (FGDs) with two groups of female supervisors and one group of male supervisors, as well as through an in-depth interview with the school principal….”

Revision:

“The participants in the qualitative study were twenty santri supervisors and one school principal. Data were collected through three focus group discussions (FGDs) with two groups of female supervisors and one group of male supervisors, as well as through an in-depth interview with the school principal. In this boarding school, supervisor is a senior student chosen by the school to take the role of supervisor. This designated santri supervisors (male and female) have at least completed senior high school from this school and they are chosen based on their high performance for the standard on religious practice and competence. Once they become supervisors, they have to live in the boarding school and are responsible to take care of 30-50 santris’, teach the Qur’an while undertaking their own activity, such as working as school staff or studying at university level.

ii. What is the source of your data collection tools?

Our response:

Thank you for your question. For the qualitative study, questions were asked based on the interview guide prepared by the first author. All interviews and FGDs were audio-recorded and transcripts were produced afterwards for further analysis. Under the data collection and analysis qualitative section (page 7, line 148-151), we have provided the descriptions of the qualitative data collection tools.

***Quantitative study***

i. What was the source of quantitative data collection tool?

Our response:

Thank you for your questions. For the quantitative study, we used four types of questionnaires adapted from studies undertaken by Khasawneh and Al-Zawahreh, Patrick, Miko *et al*., and Saffari *et al*. This description was written in the quantitative section, on data collection and analysis quantitative section (page 8, line 177-184).

ii. You mentioned the use of a Likert scale. How did you test for the internal reliability of items assessing the same variable?

Our response:

We adapted the questionnaire from Khasawneh and Al-Zawahreh which has good reliability for all constructs. In addition, we conducted content validity by asking experts and face validity by testing questionnaires on several students.

iii. How did you control for clustering at the data analysis stage?

Our response:

Thank you for your questions. We did not control for clustering at the data analysis stage because the number of individuals in the cluster was small, too small for a cluster analysis. The result will not be valid if the cluster size is small, and the number of rooms in each cluster was also small. We cannot change the cluster size because the research setting had a fixed cluster, unlike population in the community.

**Training intervention**

i. How did you ensure that training was the same for the different intervention groups?

Our response:

We appreciate your comment to clarify the intervention. In this study, we first did a training for the trainers for a duration of 8 hours. Training was given by the first author and all supervisors attended the training program. A guideline for conducting the intervention was then distributed to all supervisors to minimize variations among them. During the intervention, the first author conducted supervision by attending all intervention sessions conducted by the supervisors. Santris’ enthusiasm, consistency of supervisors in delivering training materials and training implementation were recorded during supervision by the first author

ii. Did you assess the intervention fidelity to ensure that the interventions delivered were in adherence to the protocols of the study?

Our response:

Thank you for your questions. The first author conducted supervision in all intervention sessions for the santris. Based on notes made during these observations, the supervisors delivered the training in a consistent way and the intervention was conducted according to the guidelines with a minor adjustments responding to the field conditions.

**Results**

It appears you statistically compared the outcomes within the boys groups with the outcomes within the girls group at some point. Such a comparison will require unpaired samples t-test and not a paired samples t-test.

Our response:

Thank you for raising this important comment. To clarify this point, we used the independent t-test to test the difference between gender, as stated in the narrative below table 3 (page 15-16). The paired t-test was used to measure the differences between before and after within group of interventions. We have now added few sentences to further mention the independent t-test in the data analysis section.

Original sentence (page 9, lines 177-190):

Data analysis was conducted using Stata 12 software. The variables were categorized into four levels (reaction evaluation, knowledge, behavior, and outcome) following Kirkpatrick’s training evaluation method (18). The first level was to assess reactions using a Likert scale. Numeric data was presented in a two-by-two table for each indicator and described using the means and standard deviations. The second level was to assess knowledge, which we did using paired t-tests that calculated the mean difference between pre-training and post-training intervention knowledge levels. Differences between the three interventions (video, poster, and leaflet) were tested using one-way ANOVA. The third level involved assessing differences in personal hygiene over the course of the intervention, which we tested using a paired t-test. Again, differences in hygiene between the three interventions were assessed by one-way ANOVA. Finally, the fourth level was to examine room hygiene as an outcome. Wilcoxon signed-rank tests were used to calculate the mean differences in room hygiene before and after the training, and a Kruskal-Wallis test was used to assess differences between interventions.

Revision:

“Data analysis was conducted using Stata 12 software. The analysis was carried out for each of the variables (reaction evaluation, knowledge, behavior, and outcome), following the four levels in the Kirkpatrick’s training evaluation method (18). The first level of evaluation was to assess reactions using a Likert scale. The data were presented in a two-by-two table for each indicator and described descriptively using means and standard deviations. The second level of evaluation was to assess knowledge, using paired t-tests to calculate the mean differences between pre- and post-training intervention within the group. Differences between gender were tested using an independent t-test, while differences between the three interventions (video, poster, and leaflet) were tested using one-way ANOVA, continued by the Bonferroni post hoc analysis. The third level of evaluation focused on individual behaviour, i.e. assessing differences in personal hygiene over the course of the intervention, which was tested using a paired t-test. Gender differences were tested using an independent t-test and similar to the second level of evaluation, differences in hygiene between the three interventions were assessed by applying a one-way ANOVA, continued by the Bonferroni post hoc analysis. Finally, the fourth level was evaluated by examining room hygiene as the effect. For the analysis on room hygiene (outcome), the data was not normally distributed and the number of rooms did not meet the requirement of sample size for a parametric test. Therefore, non-parametric test was used. Wilcoxon signed-rank tests were used to calculate the mean differences in room hygiene before and after the training, and a Kruskal-Wallis test was used to assess differences between interventions.”
