# Supplementary material for: Training intervention to improve hygiene practices in Islamic boarding school in Yogyakarta, Indonesia: A mixed-method study
Source: PLoS One. 2020 May 29;15(5):e0233267. doi: 10.1371/journal.pone.0233267 (PMC7259706; doi:10.1371/journal.pone.0233267)
Supplement: Attachment — Submitted filename: Comments.docx [file pone.0233267.s008.docx]

Comments:

1. In Introduction Line 78-83, please add references the authors cites?
2. Line 90-92, why the authors selected mixed method? What they reasons? Regarding previous studies in Indonesian setting.
3. In method, because just one sites study, the authors should explain their limitation for generalization?
4. In qualitative study, please explain the saturation of data? How the researchers stop their interview for saturation data?
5. In FGD, are the authors conducted separately the FGD? How they divided the group? Related to saturation data? Any data observation?
6. Please add your limitation of study?
