# Supplementary material for: Training intervention to improve hygiene practices in Islamic boarding school in Yogyakarta, Indonesia: A mixed-method study
Source: PLoS One. 2020 May 29;15(5):e0233267. doi: 10.1371/journal.pone.0233267 (PMC7259706; doi:10.1371/journal.pone.0233267)
Supplement: Attachment — Submitted filename: Response 2 to Reviewer 2_upload.docx [file pone.0233267.s009.docx]

Comments:

1. In Introduction Line 78-83, please add references the authors cites?

Our response:

Thank you for the suggestion. The statement in Introduction lines 78-83 based on the result of the preliminary study. We have now stated explicitly in the introduction:

Original sentence:

“Hygiene facilities and personal hygiene were generally poor, such as shown by poor garbage system, santri’s behavior of rarely changed their clothes and eat by hand without washing hands, and so forth. With 10-12 students in one room, low awareness about maintaining room hygiene was also reported, particularly in the girls’ room who have much more stuff.”

Revision:

“Based on the preliminary study that was conducted by first author in January 2015 at Mawar Islamic Boarding School (6), we found that hygiene facilities and personal hygiene were generally poor, such as shown by poor garbage systems, santri’s behavior of rarely changed their clothes and eat by hand without washing hands, and so forth. With 10-12 students in one room, low awareness about maintaining room hygiene was also reported, particularly in the girls’ room who have much more stuff.”

2. Line 90-92, why the authors selected mixed method? What they reasons? Regarding previous studies in Indonesian setting.

Our response:

Thank you for the question. We used the mixed-method study in order to ensure an appropriate design is used in this congregate setting. The qualitative study was first undertaken to explore perceptions and current hygiene practices among the supervisors and to identify the potential interventions. Based on qualitative findings, the interventions were developed implemented in the following quantitative study. In the quantitative study, we measured the differences in knowledge, behaviour, and outcome before and after the interventions among different interventions and according to gender. There was quite a lot of research on Islamic Boarding Schools setting in Indonesia, but specific interventions regarding hygiene practices were still quite new. The characteristics, circumstances, and facilities at each Islamic Boarding School are different, thus specific tailor-made interventions are needed. Therefore we tried it first on 1 Islamic Boarding School.

3. In method, because just one sites study, the authors should explain their limitation for generalization?

Our response:

Thank you and we agreed with your suggestion. We provide additional information regarding one site study at the limitation.

Original sentence:

“This study had limitations due to non-random allocation of participants into the cluster intervention groups, as reflected by the lower pretest scores of knowledge in the poster intervention group and lower pretest scores of personal hygiene among those in the video intervention group. Regarding the possible threat of diffusion, we were not able to fully minimize the possibilities of occurring in this study, due to arrangements in the santris’ daily activities and sleeping arrangement in this congregate boarding school setting. To reduce the risk of bias, we first applied the interventions using video, followed by posters and leaflets. The santris only watched the video during the intervention and did not have access to the files. While for the posters, they were only displayed in the santris’ rooms who belong to this inter­vention cluster. Leaflet was implemented as the last intervention because of the possibilities for the santris’ to share with others. For future research, we suggest to minimize this threat using a more rigorous study design such as the counterbalanced design.”

Revision:

“This study had limitations due to only one site study with non-random allocation of participants into the cluster intervention groups, as reflected by the lower pretest scores of knowledge in the poster intervention group and lower pretest scores of personal hygiene among those in the video intervention group. Regarding the possible threat of diffusion, we were not able to fully minimize the possibilities of occurring in this study, due to arrangements in the santris’ daily activities and sleeping arrangement in this congregate boarding school setting. To reduce the risk of bias, we first applied the interventions using video, followed by posters and leaflets. The santris only watched the video during the intervention and did not have access to the files. While for the posters, they were only displayed in the santris’ rooms who belong to this inter­vention cluster. Leaflet was implemented as the last intervention because of the possibilities for the santris’ to share with others. For future research, we suggest to minimize this threat using a more rigorous study design such as the counterbalanced design.”

4. In qualitative study, please explain the saturation of data? How the researchers stop their interview for saturation data?

Our response:

Thank you for the question. In this study, we involved 20 supervisors and one school principal. The selection of the 20 supervisors was carried out in collaboration with the school owner to appoint those who have experience in supervising new, first-grade junior high school students. Out of 45 junior high school supervisors, only 20 supervisors met the criteria. So, we interview all of the supervisors who met the criteria. The required data has been fulfilled because the participants were limited number of supervisors. The purpose of our qualitative research was to develop programs, not one of the qualitative studies tradition. For the trustworthiness, we provided summary transcripts to informants with the intention of getting feedback and confirm the information.

5. In FGD, are the authors conducted separately the FGD? How they divided the group? Related to saturation data? Any data observation?

Our response:

Thank you for the question. Data were collected through three focus group discussions (FGDs) with two groups of female supervisors and one group of male supervisors. For the FGD in the female's group, we were assisted by the chief supervisor to adjust the participant's time and split it into 2 groups. Following our statement in the previous question, we conducted FGD on all supervisors who met the criteria, then the data saturation was based on all the information we got. We did not conduct field research in this study because we have done it in preliminary studies.

6. Please add your limitation of study?

Our response:

Thank you for your comment. Our study limitation stated on the discussion section, page 20, lines 367-379 as the following:

“This study had limitations due to only one site study with non-random allocation of participants into the cluster intervention groups, as reflected by the lower pretest scores of knowledge in the poster intervention group and lower pretest scores of personal hygiene among those in the video intervention group. Regarding the possible threat of diffusion, we were not able to fully minimize the possibilities of occurring in this study, due to arrangements in the santris’ daily activities and sleeping arrangement in this congregate boarding school setting. To reduce the risk of bias, we first applied the interventions using video, followed by posters and leaflets. The santris only watched the video during the intervention and did not have access to the files. While for the posters, they were only displayed in the santris’ rooms who belong to this inter­vention cluster. Leaflet was implemented as the last intervention because of the possibilities for the santris’ to share with others. For future research, we suggest to minimize this threat using a more rigorous study design such as the counterbalanced design.”
