# Supplementary material for: Training intervention to improve hygiene practices in Islamic boarding school in Yogyakarta, Indonesia: A mixed-method study
Source: PLoS One. 2020 May 29;15(5):e0233267. doi: 10.1371/journal.pone.0233267 (PMC7259706; doi:10.1371/journal.pone.0233267)
Supplement: Attachment — Submitted filename: Reviewer Comments to Authros.docx [file pone.0233267.s002.docx]

The article will benefit by strengthening the following:

**Introduction**

Introduction well written and sets the scene, the background and introduces the problem. However, the last introductory paragraph, p.5, lines 100 – 105 which is supposed to highlight the purpose and objectives of the study is not very clear, but rather too general. The objective must stand out, and be specific and measurable. I suggest you consider phrasing it as follows:

‘To determine the effect or impact of different interventions in the overall improvement in subjects’ knowledge, behavior and outcome’

From this overall purpose, you can then deduce different objectives or hypothesis based on knowledge, behavior, etc.

With this, the study will have clear purpose and objectives. These will also inform the conclusion.

**Study site**

Page 5, lines 115-117 makes mention of ‘supervisors’. Clarity is needed on whether these ‘supervisors’ are senior students (prefects) or members of staff recruited to supervise santris.

**Study design**

The study design is well articulated in terms of mixed-methods approach. However, the quantitative part can be further strengthened. The actual design for the quantitative is ‘quasi-experimental’, because subjects were not randomly assigned to the different treatment groups. They were in their own pre-existing groups [You may consider reading Ary, D., Jacobs, L. C., Sorensen, C., & Razavieh, A. (2010). *Introduction to research in education*. Cengage Learning. 8^th^ Edition]. The ‘stepped wedge cluster prepost design’ indicated on p.6, line 127 is a kind of quasi-experimental design.

I suggest you start by indicating that your design is ‘quasi-experimental’ and discuss why you opted for it and highlight validity concerns with this type of design and how you have addressed them in this study.

p.6, lines 123-125: These are actually study objectives, they are misplaced here. Move them to objectives section I proposed earlier.

**Quantitative study**

p.8, Lines 167 reads: ‘*Four instruments were tested following the Kirkpatrick evaluation method*’. This needs clarification, were the instruments *tested* or **designed** following Kirkpatrick Model? Please clarify.

p.9, Lines 177-190 on data analysis. Relevant statistical procedures employed. However, there is need to indicate whether the assumptions for the statistics used were tested and found tenable. The assumptions includes normality, homogeneity of variance for ANOVA, more so that the sample sizes were not equal. One may assume that by using non-parametris tests like Wilcoxon signed-rank and Kruskal-Wallis tests was motivated by assumaptions not being met for parametric tests?

In using ANOVA for testing treatment effect, there are three groups (video, poster and leaflet). There is no mention of any post hoc tests to show which groups differ. In all the discussion section, authors indicate that one treatment was better than the other, but there is no mention of using post hoc tests to substantiate that. How did you know the poster and video had more impact than others without post hoc tests?

p.10, Lines 201-216: There are several validity concerns with the design approach and this is not mentioned here. Indicate how you addressed the Experimenter effects – ‘self-fulfilling prophesy’, novelty effects, and possible threat of diffusion. For the latter, the study was conducted in the school environment and it may be that students informed each other. This has a potential to negating variation.

p.12-13, Lines 242 – Commendable for using thick descriptions, the voice of the subjects is heard.

p.18, Lines 292-299: Not necessary here, it is part of literature review or introduction. Delete or carry to literature review section.

p.19, Lines 312-320: This can serve best at Literature review section or introduction. It does not serve any purpose here as it does not either corroborate or contradict anything discussed here.

p. 20, Lines 349-351: This is misplaced here. Move the two sentences to the intervention design section (p.10, lines 201-216), to justify that they were 3S.

351-352: Move to literature review section or introduction

p. 29, Line 357-359: Suggest what future designs should be put in place to address this threat (bias). e.g., counterbalanced designs

**Conclusion**

p.21, Lines 363: without explicit overall aim and objectives, it becomes very difficult to have informed conclusions. Conclusions are informed by overall aim and objectives, if these are not clear, conclusion may be also wanting. I suggest authors come up with clear aims and objectives, as suggested in my comments at the beginning.
